# Faith-Healing

**Published:** 1887-02-26

**Authors:** 


### FAITH-HEALING.

Mr. Milner Stephen is calling the attention of the world to the practice of healing the sick by faith. If Mr. Stephen performs *bonâ fide* cures we having nothing to say against him. As the Scotch have it,

> " Facts are chiels that will na' ding,

> And daur na' be disputed."

If the work of the faith-healer be real and verifiable, we shall be the very last to utter a single word of condemnation. But is it real? That is the point at issue. There are persons who dismiss the whole subject with contempt. They regard the faith-healer as a knave, and the healed as foolish dupes, and so make an end of the matter. That process has, at any rate, the merit of being short and decisive. It does not, however, quite recognise to the full the claims that the weak have upon the strong, the foolish and ignorant upon the instructed and the wise. It is highly probable, perhaps it is certain, that faith-healers are worthy only of peremptory and contemptuous dismissal from the minds of thoughtful men. But that is not the case with many of their unfortunate dupes. A poor man is blind and disqualified for work, but he cannot live except by his work or by the charity of others. The doctors have failed to cure him; they know that he is incurable. Who is to blame him if he listens to the plausible story of the man who professes to have such dealings with heaven that he can command the Divine Power to effect what the human has failed to accomplish?

To the uninstructed man there is no insuperable difficulty in a miracle. The idea of *à priori* impossibility is to him absurd. "Did not the Almighty make him," he will reason, "and did He not both make the eye and give it the power of seeing at the first? And if He could at the outset confer upon the organ the seeing power, why should He not, if He so chooses, restore to it the power which has been lost? Surely," he will argue, "it is as easy to restore a lost power as to create one which had no previous existence. Indeed, if it comes to that," he may say, "it would seem to be the easier of the two. At any rate, there is no harm in trying." Now, such a man as this does not deserve to be treated with contempt. His object is in every way laudable; and it is for the sake of him, and others like him, that we desire to give a brief and sober consideration to the subject. We place ourselves by his side to this extent, that we avow our belief in the possibility of what is called a miracle, a sign, a striking and unusual event. Miracles are not *à priori* impossible. The man who affirms that they are, affirms a principle with which true science can have no sympathy, and which is fatal to scientific progress. Hume affirmed no such principle. All that he demanded was evidence of such a character as to leave no reasonable doubt in a candid mind. Of course he tried to show that no such evidence was forthcoming. But that does not affect the logical position. We grant, then, the possibility of a miracle.

But now the sober-minded man should consider for a moment what he understands by a miracle. It is an immediate and striking intervention of the Deity. What, then, must be its characteristics? Will they partake of the nature of legerdemain? Is it probable that such an intervention will be accompanied by anything like conjuring or mystery, or arts of doubtful propriety? Will it not at least be simple, open, evident, perfect, and permanent? These are the kind of tests by which to try the so-called miracles of this or any other time. If a wonderful work has been done, it must be such that no man can deny or doubt it. The agency he may question, but not the result. If a blind man has been cured, it must be evident to all the world that he can now see. The sceptic, as well as the believer, must be compelled to admit that now the seeing faculty is perfect.

Some time ago, before Mr. Stephen was heard of in London, Mr. K., a Wesleyan, went to his medical attendant, and informed him, as he had got no benefit from medical treatment, that he was going to try the faith-healing. K. was becoming decidedly deaf. The doctor, being a man of philosophic temperament, offered no objection, but asked his patient to come and show himself when he was cured. A month or two afterwards, K. called upon the doctor on some other business. He had evidently forgotten his promise to report the result of the faith-healing. When the business was over, the doctor began to write, in order to distract K.'s attention, and so held his head that his mouth was out of K.'s line of vision. Then he said gently, but distinctly, "Is your hearing any better, Mr. K.?" No answer. A second time, and louder, came the question, "Did your grandmother take snuff, Mr. K.?" Still no reply. He then held up his head, so that K. should see his lips, and said, "How is your deafness, Mr. K.?" "Oh, I'm quite better, thank you, doctor." "Quite better! Why, I have been speaking to you two or three times, and you did not hear a word." K. persisted that he was well, and it was pathetic to see the eagerness with which he watched the doctor's lips during the rest of the conversation; and equally pathetic to find that, whenever the doctor turned his back so that the movements of his lips could not be seen, poor K. could not hear a single word spoken in ordinary tones. As a matter of fact, he was not a whit improved, though he was honestly prepared to assert that he was quite well.

Now here was a sincere dupe—a man of undoubted character and of some education. How many converts might not such a man make? He, however, had paid no fees for his cure. It is probable that those who had operated upon him were as much deceived as himself. And this is surely an important question to ask of any man who professes to heal by faith: Does he gain wealth by the exercise of his so-called power? If he does, should not that be sufficient to discredit him at once and for ever in the eyes of reasonable people, and especially Christian people? Is it conceivable that Almighty God will work miracles upon one man in order to fill the pockets of another? Is not the idea shocking and monstrous? Is there a single miracle recorded, either in the Old or New Testament, by which the worker gained pecuniary benefit? When the servant of one of the prophets took gold from the cured Syrian, the prophet denounced upon him the most terrible punishment. "The leprosy of Naaman shall cleave unto thee;" and he "went out from his presence a leper white as snow." The very same spirit is everywhere manifest in the New Testament. When miracles were wrought in ancient times there was no trafficking for profit. The miracle was a sign to mankind that a divine presence was about them for great purposes.

That the so-called miracles of modern times should be so readily believed in, even by persons of education, is a convincing proof that, in spite of the advances of science among the few, the many are still in a condition of intellectual childhood, and are as capable of falling back into the most stupid and abject superstitions as any of the savage races to whom education is unknown. Christianity and science concur in this, that whatever fails to commend itself to candid reason and true simplicity of heart, cannot be worthy of the attention of honest and thoughtful men.

